# A Priori-Based Subarray Selection Algorithm for DOA Estimation

**DOI:** 10.3390/s20164626

**Published:** 2020-08-17

**Authors:** Linghao Zeng, Guanghua Zhang, Chongzhao Han

**Affiliations:** Ministry of Education Key Laboratory for Intelligent Networks and Network Security, School of Automation Science and Engineering, Xi’an Jiaotong University, Xi’an 710049, China; zenglh@stu.xjtu.edu.cn (L.Z.); czhan@xjtu.edu.cn (C.H.)

**Keywords:** sparse array synthesis, DOA estimation, low-rank matrix approximation

## Abstract

A finer direction-of-arrival (DOA) estimation result needs a large and dense array; it may, however, encounter the mutual coupling effect, which degrades the performance of DOA estimation. There is a new approach to mitigating this effect by using a nonuniform array to achieve DOA estimation. In this paper, we consider a priori DOA estimation, which is easily obtained from tracking results. The a priori DOA requires us to pay close attention to the high possibility of where the DOA will appear; then, a weight according to the prior probability distribution of DOA is added to each direction, which leads the sensing matrix of DOA estimation to be near low-rank. Thus, according to the low-rank matrix approximation theory, an optimal low-rank approximate matrix is obtained and an algorithm is proposed to select the elements of the original array according to right singular vectors of the approximate matrix. After that, the impacts of different weights are analyzed, and a mixed weight is presented which has flexibility for common use. Finally, a number of numerical simulations are carried out, and the results verify the effectiveness of the proposed methods.

## 1. Introduction

Data acquisition from an array sensor is a kind of spatial signal sampling which needs to satisfy the Shannon–Nyquist sampling theorem. Uniform linear arrays (ULA) with equal intervals are therefore used in many practical applications. Meanwhile, DOA estimation is an important branch of array signal processing and has a wide range of applications [1,2,3]. In order to achieve higher resolution and antinoise capability, several array elements are needed to achieve a larger aperture. This will not only increase the cost of the sensor but also degrade the array performance due to the mutual coupling effect of the array elements.

To solve these problems, according to the nonuniform sampling theory [4], small, elaborately arranged nonuniform arrays can be used to achieve similar performance to a large uniform array. Sparse array synthesis, therefore, has become an important research direction and has attracted the attention of many researchers. Some computer-based intelligent optimization methods are suitable for solving these problems, including the genetic algorithm [5], differential evolution algorithm [6], particle swarm optimization [7], etc. These algorithms are solved iteratively, and the calculation cost is large. The coprime array configurations for DOA estimation are analyzed in [8,9]; these research works are concerned with the degree of freedom in DOA estimation. In [10,11], a matrix pencil method (MPM) is proposed to reduce the number of sensors in linear arrays, and the used algorithms show better performance in terms of convergence speed and accuracy. In [12], a cognitive radar antenna selection method based on deep learning is presented. The authors construct a deep neural network with convolutional layer as a multiclass classification framework. This method is effective in one-dimensional scenarios, but its performance degrades for two-dimensional cases.

In the past decade, the sparse signal sampling technique of compressed sensing (CS) is presented in [13]. This relaxes the limitation of the Shannon sampling theorem when the original signal is sparse and allows us to reconstruct the sparse signal with fewer measurements [14]. In [15], the authors propose a method that equates the beam-forming problem of the array antenna to the optimization problem of solving the sparse signal vector, using the FOCUSS algorithm to quickly and accurately obtain an array with the maximum sparsity, element position and excitation amplitude.

The problem studied in this paper is that, in a target tracking scenario, the prediction of target position is used a priori to select the elements of a large-scale array and obtain a sparse subarray with similar performance to the original array. This is similar to the sparse array synthesis mentioned above, but the difference is that we consider the sparse array synthesis from the perspective of the application of target tracking.

Target tracking is a dynamic process. At each moment, the echo from the target is obtained through a sparse array, the bearing information of the target is estimated through a DOA estimation algorithm and the estimation and prediction of the target position and velocity are calculated by a target filter. In this process, we can use the a priori prediction of the target orientation to obtain a sparse subarray that changes with time. In order to realize the changing of the subarray, in this paper, the sparse subarray consists of the elements in a large-scale array; thus, “subarray selection” is the focus of this paper.

In addition, the aim of this paper is not simply to optimize array performance but to optimize the overall target tracking performance. Specifically, this means that the error of DOA estimates by using the sparse subarray is similar to by the original larger array. This contrasts with the performance index of traditional array synthesis. The algorithm for DOA estimation, therefore, needs to be determined first. Some classical super-resolution algorithms—e.g., MUSIC [16] and ESPRIT [17] —need multisnapshots for subspace decomposition, and their performances may degrade when the echoes of targets are correlated. There are also some deep learning-based algorithms: A stacked autoencoder-based method is introduced in [18]; A DOA estimation algorithm based on deep neural network is proposed in [19]; A deep learning based scheme for achieving super-resolution DOA estimation and channel estimation in the massive multiple-input multiple-output system is presented in [20]. These methods need a large number of samples for training neural network.

In contrast to the above algorithms, compressed sensing-based DOA estimation (CS-DOA) algorithms only use a single snapshot, and there is no correlation problem of the echoes of targets; we therefore adopt CS-DOA algorithms in this paper [21,22,23]. Since some CS-DOA algorithms require the columns of the sensing matrix (flow pattern matrix) to be unified, the excitation of each element in the sparse subarray is designed to be the same in this paper. Moreover, the performance of the compressed sensing reconstruction algorithms mainly depends on the column coherence of the sensing matrix. The column coherence, therefore, is an important index for sparse subarray selection [24,25].

The specific idea of this paper is to use the target position prediction provided by the target filter a priori to select the subarray. In practical applications, especially in air traffic control, radar target tracking, etc. [26,27], the a priori target position is easily obtained. The prediction of the targets shows where the targets are most and least likely to appear. We should naturally pay increased attention to the area with the highest probability of appearance when sensor resources are limited. In order to reflect this difference in attention, we add a series of weights to each direction of the surveillance area according to the prior probability of the target position to obtain a weighted sensing matrix.

Similar to the method in [10,11], we perform singular value decomposition (SVD) on the weighted sensing matrix to obtain a low-rank approximation matrix [28]. We then find the best matching combination of the rows in the original sensing matrix according to the feature space consisting of the right singular vector of the low-rank matrix. The array elements corresponding to these rows are the sparse subarray that we need.

Since different weights bring different results, in the above method, we need to determine the weight added to the sensing matrix. Considering that the a priori target positions obtained by the target filter usually have the form of a Gaussian function, a direct idea is to apply it directly on the sensing matrix as the weight. Therefore, after analyzing and comparing the influence of the Gaussian weight and rectangular weight on the eigenvalue of the weighted sensing matrix and the subarray selection algorithm, we propose a type of weight to achieve the purpose of performance balance.

The main contributions of this paper are as follows. Firstly, by analyzing the influence of different weights on the eigenvalue of the weighted sensing matrix, we present a method for generating a performance-balanced weight, which makes the proposed algorithm more applicable to the case where the a priori target position includes deviation. Secondly, according the low-rank matrix approximating theory, we find the optimal approximate matrix of the weighted sensing matrix and analyze the rank of the optimal approximated matrix on different conditions, such as width of the weight and total element number of the original array. Thirdly, we propose a subarray selection algorithm by utilizing the correct singular vectors as the features to find required elements in the original array; the simulation results show the effectiveness of the proposed algorithm.

The paper is organized as follows. Section 2 introduces the discrete model of the CS-based single snapshot DOA estimation and the influence of column coherence in DOA estimation. In Section 3, we analyze the effect of different weights on the singular values of the weighted sensing matrix though the SVD method and present an analysis of the rank of the low-rank approximate matrix of the weighted sensing matrix. The proposed subarray selection algorithm is listed in Section 4, and the numerical simulations are given in Section 5. Finally, the paper is summarized in Section 6.

## 2. Compressed Sensing-Based Single Snapshot DOA Estimation

### 2.1. Mathematical Model

Before discussing subarray selection, it is necessary to introduce the mathematical model of far-field single snapshot DOA estimation. Here, we suppose there is a measurement system with one transmitter and a receiver array. A unit-energy rectangular pulse waveform is periodically transmitted to a particular two-dimensional area. The transmitted signal is reflected by the target, and then the echo impinges on the receiver array. It should be noted that the case of secondary echoes is not considered here for simplicity.

Without loss of generality, suppose the array receiver consists of *M* identical omnidirectional sensors whose positions are indicated by $p=[p_{1},\cdots ,p_{M}]$ where $p_{i}={\left[p_{x,i},p_{y,i}\right]}^{T}$. Here, *p* can be arbitrary and can be given by the actual situation or the user’s requirements. The receiver samples the echo signal periodically. When ignoring the Doppler effect, the sampled echo signal is represented by

(1) $$y=\sum_{q=1}^{Q}a\left(\theta_{q}\right)x_{q}+e,$$

where *Q* is the total number of targets, *e* is the noise component on the receiver, *x* is the complex amplitude of the target, $\theta_{q}\in \left(-\pi /2,\pi /2\right]$ is the direction of the $q_{th}$ target and $a(\theta_{i})$ is the corresponding steering vector for the received array with its form of

(2) $$a\left(\theta \right)=[1,\exp\left(-i\frac{2\pi r_{p,1}}{\lambda }\sin\left(\theta -\theta_{p,1}\right)\right),\cdots ,\exp\left(-i\frac{2\pi r_{p,M}}{\lambda }\sin\left(\theta -\theta_{p,M}\right)\right)],$$

where λ is the wavelength of the carrier wave, ${\left(\cdot \right)}^{T}$ represents the transposition operation, “i” is the imaginary unit, $r_{p,i}=∥p_{i}∥$ and $\theta_{pi}=\arcsin\left(p_{y,i}/r_{p,i}\right)$. We set the normal direction of the sensor array as θ=0.

According to the discrete model of single snapshot DOA estimation in [29], the whole surveillance area of (−π/2,π/2] is discretized into *N* discrete angle bins, and it is denoted by $\left\{\theta_{1},\cdots ,\theta_{N}\right\}$; then, the Equation (1) can be rewritten as

(3) y=Ax+e,

where $A=\left[a\left(\theta_{1}\right),\cdots ,a\left(\theta_{N}\right)\right]$ is called the sensing matrix in terms of compressive sensing (or the manifold matrix) and $x={\left[x_{1},\cdots ,x_{N}\right]}^{T}$ represents the complex amplitudes from the corresponding discrete angle bins. Equation (3) is a standard form in compressive sensing theory, and the DOA estimation can be solved by the following optimization problem:

(4) $$\min_{x}\;\;{∥x∥}_{0},\;\;s.t.\;\;y=Ax+e,$$

where ${∥\cdot ∥}_{0}$ denotes the number of nonzero elements of a vector.

### 2.2. Coherence of Sensing Matrix

In many applications for compressed sensing-based DOA estimation, the algorithm performance depends on the coherence of the sensing matrix [30]. The coherence is described by the inner-product of two columns in sensing matrix. From Equation (2), the coherency coefficient between $a(\theta_{i})$ and $a(\theta_{j})$ is given by

(5) $$c_{\theta_{i},\theta_{j}}=a^{H}\left(\theta_{i}\right)a\left(\theta_{j}\right)=\sum_{m=1}^{M}\exp\left(-i\frac{2\pi r_{p,m}}{\lambda }\left(\sin\theta_{i}-\sin\theta_{j}\right)\right),$$

where ${\left(\cdot \right)}^{H}$ denotes the conjugate transpose operation. We can thus obtain a coherence function of direction $\theta_{i}$, defined on θ∈(−π/2,π/2]):

(6) $$c_{\theta_{i}}\left(\theta \right)=\sum_{m=1}^{M}\exp\left(i\frac{2\pi r_{p,m}}{\lambda }\left(\sin\theta -\sin\theta_{i}\right)\right).$$

The coherence function $c_{\theta_{i}}\left(\theta \right)$ is similar to the antenna pattern. According to the array signal processing theory, the aperture of the array determines the width of the main lobe of curve of $c_{\theta_{i}}\left(\theta \right)$, and the element interval space of a ULA determines the level of the side lobe.

In a high signal-to-noise (SNR) environment, noise makes the estimated angle deviate from its true value to its surroundings. The covariance of the error is proportional to the noise level and the proportion coefficient is related to the width of the main lobe of coherence curve.

Moreover, when the noise is greater than a thresholding value, the DOA estimation error is no longer proportional to the noise level, and the estimated angle may appear in the area corresponding to the side lobe of the coherence curve; i.e., an outlier. Therefore, we use the term “noise immunity” to measure the thresholding value of noise. According to [31], a higher side lobe level means a lower thresholding value of noise or, in other words, a low capability for noise immunity.

According to the discrete model mentioned in Section 2.1, we can use a coherence vector to characterize the response of an array to the direction $\theta_{i}$, which is defined by

(7) $$g\left(\theta_{i}\right)=A^{H}a\left(\theta_{i}\right)={\left[c_{\theta_{i}}\left(\theta_{1}\right),\cdots ,c_{\theta_{i}}\left(\theta_{N}\right)\right]}^{T}.$$

where $g(\theta_{i})$ is a sample of $c_{\theta_{i}}\left(\theta \right)$ at the discrete angles corresponding to the columns of *A*. The Gram matrix of the sensing matrix, therefore, is able to characterize the DOA estimation performance of the array, and it is given by

(8) $$G=A^{H}A=\left[g\left(\theta_{1}\right),\cdots ,g\left(\theta_{N}\right)\right].$$

## 3. Low-Rank Approximation of Weighted Sensing Matrix

The aim of this paper is to find a sparse subarray with a similar DOA estimation performance to the original large-scale array by utilizing the prior information of DOA.

### 3.1. Weighted Sensing Matrix

The prior information about DOA is usually taken from the prediction of a target filter which gives it a probability density function. For the case of multitarget tracking, if the prediction of each target is given by $p_{a,q}\left(\theta \right)$, respectively, the sum of the predictions is denoted by $p_{a}\left(\theta \right)$. It should be noted that $p_{a}$ is not a PDF when Q>1 and is called target intensity in the research into multitarget filters [32].

When target tracking is stable, the prior information is reliable for indicating the possibility of the target appearing in different directions. This inspires us to pay different levels of attention to these directions. Then, we design a weight of *w* for each column in *A* to represent it. If we assume $w={\left[w_{1},\cdots ,N\right]}^{T}$ and W=diag(w), the weighted sensing matrix is represented by

(9) $$A_{W}=AW.$$

Different weights may lead to different results. Here, we give three basic type of weight: the uniform weight, which corresponds to the original sensing matrix, which means no prior information; the Gaussian weight; and the rectangular weight.

For the sake of description, we set the maximum value of each weight to one. After that, the rectangular weights are all binary values, and the Gaussian weights equal a Gaussian function divided by its maximum, keeping the maximum of the weight equal to one. It is worth mentioning that it is the type of weight rather than the value that plays an important role in the following analysis.

Singular value decomposition (SVD) is an appropriate tool for analyzing a nonsquare matrix. We assume the weighted sensing matrix has an SVD denoted by

(10) $$A_{W}=U\Sigma V^{H},$$

where *U* is a unitary matrix of order *M*, *V* is a unitary matrix of order *N* and Σ is a M×N-dimensional matrix [33]. The diagonal elements of Σ are the singular value of *A*, and the other elements are equal to zero.

From Section 2.1, we know *A* is full row-rank. Especially for a ULA, the *M* mutually orthogonal columns in *A* can be found by solving $a^{H}\left(\theta_{i}\right)a\left(\theta_{j}\right)=0$, and their solution is given by

(11) $$\cos{\left(\theta_{i}\right)}^{T}-\cos\left(\theta_{j}\right)=2n/M$$

for n=1,⋯,M.

**Figure 1:**
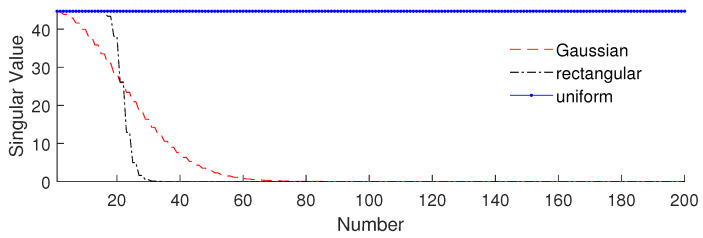
The singular values of different weighted sensing matrices.

We will focus on the effect of the weight on the singular value of sensing matrix. Figure 1 shows the singular value of different weighted sensing matrices: the blue dotted line is the original sensing matrix (uniform weight), the black dashed–dotted line corresponds to a rectangular weight with the width of 200 and the red dashed line corresponds to a Gaussian weight with a standard deviation of 100.

It is found that the shapes of singular values in descending order shown in Figure 1 are similar to the shapes of their weight. The singular values of the original sensing matrix are all the same and equal to $\sqrt{N}$, and the singular values of rectangular type scan be clearly divided into two parts: the values of the smaller part are all near zero, and those of the larger part have the same value as $\sqrt{N}$—they are of Gaussian type and have a similar shape to the folded normal distribution, with most of them being near to zero. In general, the weighted sensing matrix is near low-rank when the weight is concentrated.

**Figure 2:**
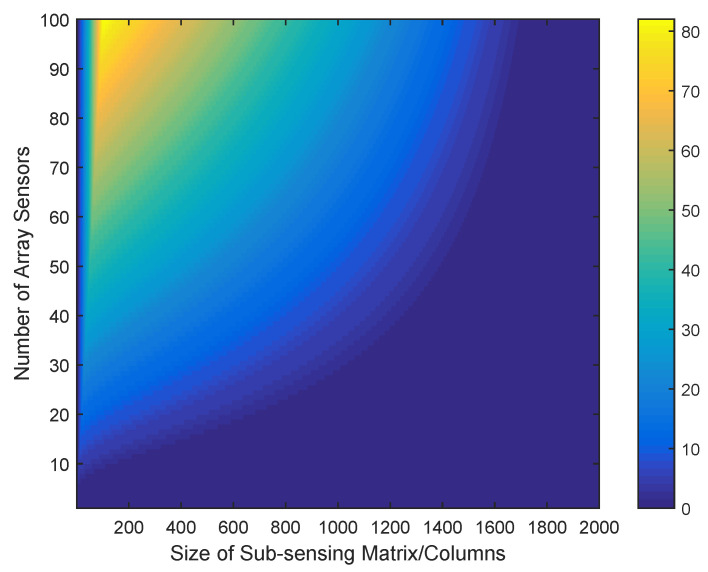
The number of smaller singular values of the sensing matrix with different weight widths versus array sensor numbers.

In the following part of this section, the effect of the weight concentration on the low-rank feature of the weighted sensing matrix is analyzed. Given that the resulting rectangular weight is similar to the Gaussian type, we choose the rectangular weight as representative for convenience. Figure 2 shows the number of smaller singular values of $A_{W}$, which are judged by a threshold value of $10^{-8}$, on the different conditions of widths of weights and numbers of array elements. The x-axis is the width of the rectangular weight, where the interval of the discrete angle is $\arcsin(10^{-3})$, and the y-axis is the number of elements used in ULA with the interval space of half a wavelength. We find that the weighted sensing matrix can be approximated by a low-rank matrix without an observable error for most conditions.

### 3.2. Low-Rank Matrix Approximation

On account of the large proportion of singular values which are almost zero, the weighted sensing matrix can be approximated by a low-rank matrix denoted by $A_{L}$ [34]. For convenience of description, the number of larger singular values of $A_{W}$ is assumed to be *K*. The low-rank matrix approximation of $A_{W}$ is therefore represented by

(12) $$\begin{array}{cc} \min_{A_{L}} & {∥A_{W}-A_{L}∥}_{F}, \end{array}$$

(13) $$\begin{array}{cc} s.t. & \mathrm{rank}(A_{L})=K, \end{array}$$

where ${∥\cdot ∥}_{F}$ is the Frobenius norm of a matrix.

According to [28], the optimal low-rank approximating matrix is given by

(14) $$A_{L}=U_{K}\Sigma_{K}V_{K}^{H},$$

where $\Sigma_{K}$ is a diagonal matrix of order *K* which consists of the larger *K* singular values of $A_{W}$, and $U_{K}$ and $V_{K}$ are the corresponding left and right singular vectors.

The factor of *K* is significant in low-rank matrix approximation for determining the rank of $A_{L}$. In fact, *K* depends on a subjective threshold value of τ, by which the definition of a small singular value can be judged. For the weighted sensing matrix, τ can be chosen according to Figure 1. In the case of rectangular weight, the selection range of τ is small, due to the fact that there is an obvious difference between the larger and the smaller singular values. In the case of a Gaussian weight, however, there is a large range for selection, because there is a smooth transition between larger and smaller singular values.

## 4. Proposed Algorithm

### 4.1. Subarray Selection Algorithm

The final goal is to find a subarray which has a similar performance in terms of DOA estimation to that of the original array. Because the subarray is a part of the original array, we can use a vector of *s* to describe which element in the original array is selected. If it has $s={\left[s_{1},\cdots ,s_{M}\right]}^{T}$ and $s_{i}\in 0,1$, the corresponding subarray can be represented by

(15) $$A_{S}=SA,$$

where S=diag(s).

Meanwhile, according to the discrete model of DOA in Section 2.2, in an ideal case, the sensing matrix of the subarray satisfies

(16) $$\begin{array}{c} A_{W}^{H}A_{W}=A^{H}SA_{W} \end{array}$$

(17) $$\begin{array}{cc} \Rightarrow & V\Sigma^{H}\Sigma V^{H}=A^{H}SU\Sigma V^{H} \end{array}$$

(18) $$\begin{array}{cc} \Rightarrow & \Sigma^{H}\Sigma =V^{H}A^{H}SU\Sigma \end{array}$$

If we use $A_{L}$ to approximate $A_{W}$, consider Equation (14); we obtain

(19) $$U_{K}\Sigma_{K}=SAV_{K}.$$

Let $B=U_{K}\Sigma_{K}$, $C=AV_{K}$; Equation (19) can be rewritten as

(20) B=SC,

where $C_{i,j}$, the element of *C*, denotes the inner product of the $i_{th}$ row of *A* and the $j_{th}$ column of $V_{K}$.

In order to make Equation (20) true, both left and right sides must first have the same rank. This means that rank(SC)=K while rank(B)=K; thus, rank(S)≥K. Meanwhile, considering that there need to be as few used elements as possible, it has rank(S)=K. Consequently, the effect of *S* is the selection of *K* rows in *C*, and the matrix of SC can be then represented by a row-reduced square matrix of $\bar{C}$.

We can use the equivalent form of Equation (20) as below:

(21) $$\begin{array}{c} B^{H}B=C^{H}S^{H}SC \end{array}$$

(22) $$\begin{array}{cc} \Rightarrow & \Sigma_{K}^{H}\Sigma_{K}={\bar{C}}^{H}\bar{C} \end{array}$$

(23) $$\begin{array}{cc} \Rightarrow & \Sigma_{K}=\bar{C}. \end{array}$$

Due the nonideal conditions in practice, Equation (23) does not always hold. We need to find an approximate constraint:

(24) $$\min_{S}{∥\Sigma_{K}-\bar{C}∥}_{F},$$

where *S* is implicit in $\bar{C}$. Due to the mismatch between *A* and $V_{K}$, it always has $C_{i,j}<=\Sigma_{K,ii}$, where $\Sigma_{K,ii}$ is the diagonal element in the diagonal matrix $\Sigma_{K}$.

We present an algorithm for solving the optimization problem mentioned in Equation (24), which is shown in Algorithm 1.

**Algorithm 1:** Subarray element selection

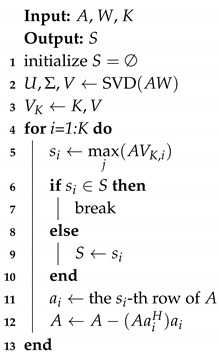



According to each orthogonal vector in $V_{K}$, the most relevant row $A_{i}$ is found. After that, the part of *A* orthogonal to $A_{i}$ is calculated in order to reduce the correlation between $A_{i}$ and the other rows; i.e., to make the nondiagonal elements smaller. Finally, *K* rows of *A* are obtained, and the corresponding sensors constitute the needed subarray.

### 4.2. Design of Weight

According to the derivation of the proposed algorithm, there are two kinds of approximation errors: the first one, which we call truncation error, arises in the process of using $A_{L}$ to approximate $A_{W}$, which depends on the sum of unselected singular values; the second one, which is called mismatch error, arises in the selection process corresponding to Equation (24), which depends on the mismatch of the correct singular vectors between *A* and $A_{W}$.

Through the analysis in Section 3.1, it is known that the weight affects the singular value of the weighted sensing matrix and the corresponding singular vectors. Consequently, we need to analyze the type of weight which causes the second approximation error.

From Figure 1, we can see that the rectangular weight added to the sensing matrix is equivalent to the deletion of some columns corresponding to where DOA will not appear. This means that the singular vectors of the weighted sensing matrix are similar to the original sensing matrix. Thus, the mismatch error is smaller and the main diagonal element in $\bar{C}$ is larger.

Nevertheless, there are still some problems regarding the rectangular weight. Because its larger singular values almost have the same value, it is difficult to select the moment when it is required that *K* should be less than the number of larger singular values. The disorder of singular values may cause some important singular vectors to be deleted.

In contrast, the Gaussian weight leads to inequality on the columns in *A*, and this may cause the mismatch of singular vectors to become worse. However, it makes the singular values ordered for the same reason.

In order to make a trade-off between these two weights, we suggest a mixed weight which is easily generated and has a balanced performance. Assuming the prior information of each DOA is given by a Gaussian distribution, we first choose a thresholding of probability denoted by $\tau_{p}$. We then generate the weight with the following equation:

(25) $$w\left(\theta \right)=\left\{\begin{array}{cc} 1, & p\left(\theta \right)\geq \tau_{p}\\ \frac{p\left(\theta \right)}{\tau_{p}}, & p\left(\theta \right)<\tau_{p} \end{array}\right.$$

For instance, supposing *x* subjects with a normal distribution of $N(0,\sigma^{2})$, we can choose $\tau_{p}=p\left(x=\sigma \right)$; thus, the probability of DOA appearing in the rectangular part is 68.27%. We can also choose a lower threshold value for a larger coverage, but this leads a higher requirement of array sensors.

The weight-generating algorithm is then summarized in Algorithm 2.

**Algorithm 2:** Weight generating algorithm

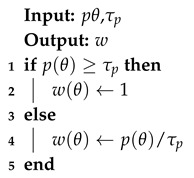



## 5. Numerical Simulation

In this section, a number of numerical simulations are carried out in different conditions to illustrate the effectiveness of the presented method by comparing it with the FOCUSS and MPM methods. It is known that the MPM method calculates the position of array elements based on the generalized eigenvalue method, and the alternate array element position cannot be specified before calculation. The MPM method is not actually suitable for the work in this paper; therefore, the simulation below only lists the results of this method for comparison with the other methods without further analysis.

In these simulations, the signal sources are from two separated far-field moving targets. The surveillance area from −π/2 to π/2 is divided into 2000 angle bins according to an equal sine difference. Each signal with its amplitude of 1 is received by the array sensors. The demanded number of subarray elements is given by K=20.

In order to achieve a fair comparison of different arrays, the noise of each array element is employed, which is complex Gaussian white noise. Both real and imaginary parts of the noise obey the Gaussian distribution given by $N(0,\sigma_{e}^{2})$. The DOAs are then estimated by the modified orthogonal matching pursuit (m-OMP) algorithm which employs the expectation maximization (EM) method to eliminate the interaction between DOAs [35].

The error of DOA estimation is usually measured by the difference between the true value and the estimated value, which is sufficient for the single-DOA case but not for the multi-DOA case. Meanwhile, the compressed sensing algorithm may cause additional outliers when the noise level is greater than $\tau_{p}$, determined by the side-lobe level. Consequently, we need a metric which is able to measure both the angle error and the number error simultaneously. Therefore, the “optimal subpattern assignment” (OSPA) method proposed in [36] is adopted to measure the DOA estimation error in this paper. It is assumed that $\Theta_{T}=\left\{\theta_{1},\cdots ,\theta_{Q}\right\}$ denotes the true DOAs and $\Theta_{E}=\left\{{\hat{\theta }}_{1},\cdots ,{\hat{\theta }}_{Q_{E}}\right\}$ denotes the estimated DOAs. When $Q_{E}\geq Q$, the definition of the OSPA distance is given by

(26) $$d_{c,p}^{OSPA}\left(\Theta_{E},\Theta_{T}\right)={\left(\min_{\mu \in \Pi_{n}}\frac{1}{Q}\sum_{i=1}^{Q_{E}}d_{c}{\left({\hat{\theta }}_{i},\theta_{\mu (i)}\right)}^{p}+\frac{c^{p}}{Q}\left(Q-Q_{E}\right)\right)}^{1/p},$$

where *p* is the order of the OSPA distance, *c* is the cut-off parameter, $\Pi_{n}$ denotes all the combinations of {1,⋯,Q}, and

(27) $$d_{c}\left({\hat{\theta }}_{i},\theta_{\mu (i)}\right)=\min\left(c,\left|{\hat{\theta }}_{i}-\theta_{\mu (i)}\right|\right).$$

If $Q_{E}<Q$, it has $d_{c,p}^{OSPA}\left(\Theta_{E},\Theta_{T}\right)=d_{c,p}^{OSPA}\left(\Theta_{T},\Theta_{E}\right)$.

There is a simple explanation of the OSPA metric. The right side of Equation (26) includes two parts: the first item represents the estimation error of the angle, and the maximum angle error of each target is limited by *c*; the second item denotes the error in the estimated number of targets, whose value is equal to $c|Q-Q_{E}|$. The parameters of *c* are suggested to be an upper bound of error, and here we give c=10 and p=1.

### 5.1. Comparison of Runtime

The methods we proposed are compared with the FOCUSS method proposed in [15] and the MPM method proposed in [10]. Due the fact that the FOCUSS and the MPM methods are simply array synthesis methods, it is necessary to give them an object pattern. According to the Section 2.2, we use the coherence curve of a weighted sensing matrix with a rectangular weight as the object pattern.

The runtime of each method under different conditions is given in Table 1 and Table 2, which are the average of 100 trials. The simulation environment is as follows: Matlab R2019b, Windows 10 64 bit, Intel Core i5-9400f CPU 2.90 GHz, RAM 16.00 GB.

It is worth noting that the effective widths of the different types of weight are not the same even if they are according to the same prior distribution. The effective width is defined as the size of the parts which have $w_{i}>10^{-3}$. For instance, if the prior distribution is given by $N(1000,50^{2})$, the effective widths of the narrow rectangular weight, wide rectangular weight, Gaussian weight and mixed weight are 101, 201, 371 and 385, respectively.

It is found that our proposed methods and the MPM method are all based on the SVD technique, which requires a large amount of computing time. Meanwhile, the computing time increases with the effective width and the number of alternative elements. Moreover, due to the FOCUSS method being an iterative algorithm, its computing time is greater than the others.

### 5.2. Simulation 1

Simulation 1 is carried out to analyze the influence of different weights on the subarray selection and the performance of DOA estimation. The original array is a ULA with M=100 elements assigned along the x-axis with an interval of half a wavelength. The number of used elements in array is fixed at K=20 while the width of weights is variant. The prior PDFs of two DOAs are given by the normal distributions denoted by $p_{a,1}=N\left(500,\sigma_{p}^{2}\right)$ and $p_{a,2}=N\left(1500,\sigma_{p}^{2}\right)$, respectively, and the true positions of these DOAs are also generated by the above PDFs; i.e., the prior information is reliable. The rectangular, Gaussian and mixed weights are generated according to the methods mentioned in the above section, and the corresponding subarrays are then obtained.

**Figure 3:**
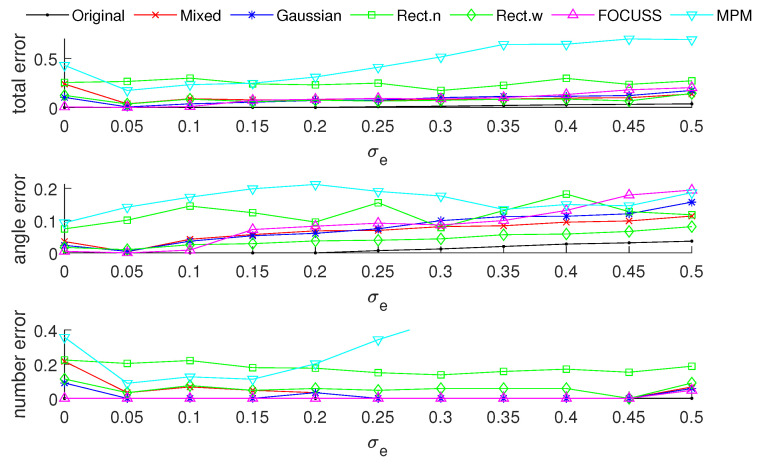
Direction-of-arrival (DOA) estimation error in the optimal subpattern alignment (OSPA) distance via different arrays based on different weights and uniform linear arrays (ULAs) when K=20; the prior information of DOA is reliable and $\sigma_{p}=30$.

**Figure 4:**
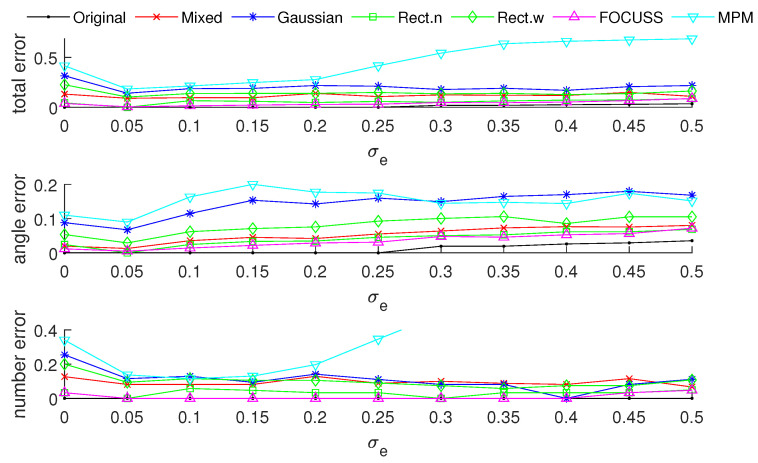
DOA estimation error in the “optimal subpattern assignment” (OSPA) distance via different arrays based on different weights and uniform linear arrays when K=20; the prior information of DOA is reliable and $\sigma_{p}=50$.

Figure 3 and Figure 4 show the DOA estimation error in terms of the OSPA distance via different arrays when the standard deviations of the prior PDFs are given by $\sigma_{p}=30$ and $\sigma_{p}=50$. The x-axis is the standard deviation of noise on each array element, and the y-axis is the DOA estimation error. The upper subfigure is the total error in terms of the OSPA distance, and the middle and lower subfigures correspond to the angle error and number error, respectively. All the results are the root mean squared error (RMSE) of 100 Monte-Carlo trials.

The blue “·” shows the error of the original array, which is considered as a reference of the DOA estimation performance; the red “×” corresponds to the subarray generated by the mixed weight, and $\tau_{p}=p\left(\sigma_{p}\right)$; the blue “∗” is the result of the subarray generated by the Gaussian weight; the green “□” and “⋄” are the subarrays generated by the rectangular weights, with widths of $2\sigma_{p}$ and $4\sigma_{p}$, respectively; the magenta “Δ” shows the error of the subarray generated by the FOCUSS algorithm; and the cyan “∇” denotes the result of the subarray generated by the MPM method.

**Figure 5:**
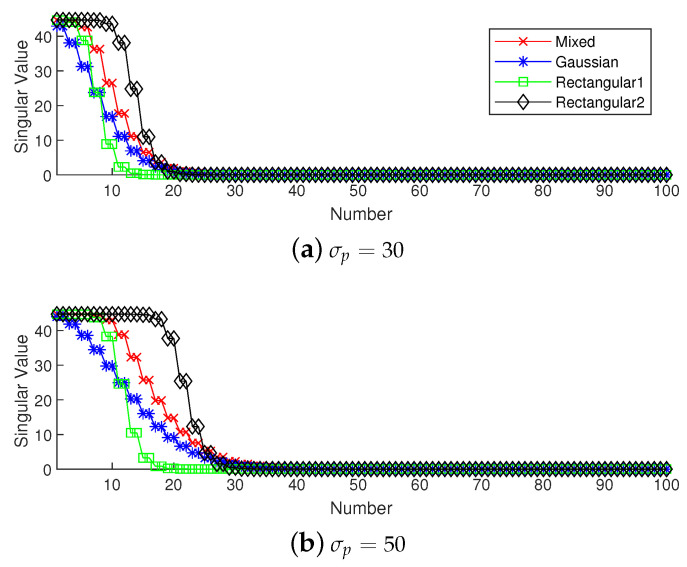
Singular value spectrum of different weighted sensing matrices with weight widths given by (**a**) $\sigma_{p}=30$; (**b**) $\sigma_{p}=50$.

To analyze the influence of different widths of weights, we first show the singular value spectrum of different weighted sensing matrices with different weight widths in Figure 5. Figure 5a shows the condition of $\sigma_{p}=30$, which corresponds to the simulation result shown in Figure 3. Due to K=20, the main features, defined as the singular vectors corresponding to the larger singular values, are all chosen by every subarray. This scenario corresponds to the case in which the required number of array elements is sufficient. In this case, the truncation error of each array is small, and the main contribution of error depends on the mismatch error. From the view of angle error, the subarray corresponding to a narrow rectangular weight, therefore, has the worst performance in our proposed methods in terms of including the fewest features; on the contrary, the wide rectangular weight has best performance in our proposed methods in terms of containing the most features, and the Gaussian weight, the mixed weight and the FOCUSS method have moderate performance. From the view of number error, due to the orderless nature of the singular vectors, the number error of the rectangular weights is larger than the other methods. All the methods except the narrow rectangular weight have a similar total performance because their boundaries of large singular values are all near 20 in the singular value spectrum.

**Figure 6:**
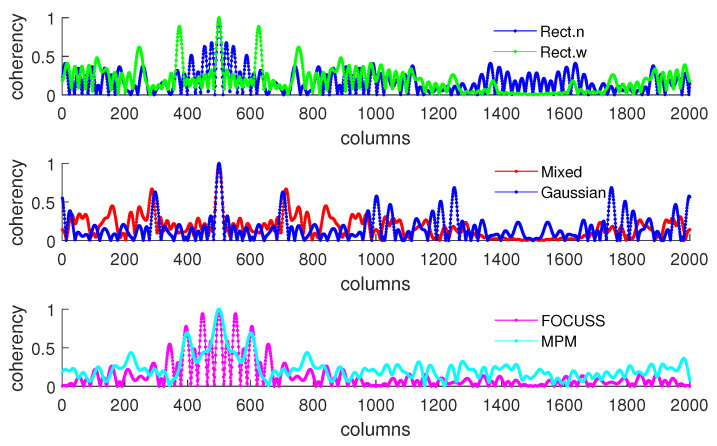
Coherence curve of each subarray generated in Simulation 1 at direction θ=π/6 when $\sigma_{p}=30$.

**Figure 7:**
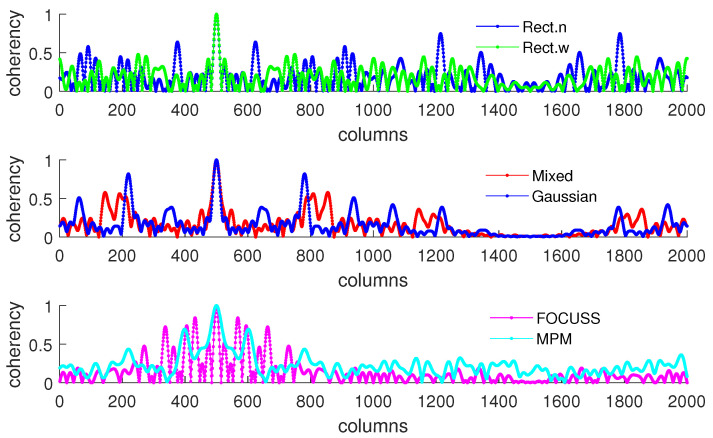
Coherence curve of each subarray generated in Simulation 1 at direction θ=π/6 when $\sigma_{p}=50$.

Figure 5b shows the condition of $\sigma_{p}=50$ which corresponds to the simulation result shown in Figure 4. This scenario corresponds to the case in which the required number of array elements is insufficient. In this case, the truncation error of the wide rectangular weight increases rapidly, which causes the angle error to become large. Due to both the truncation error and the mismatch error increasing, the performance of the Gaussian weight has the worst performance. The narrow rectangular weight and FOCUSS method both exhibit similar better performance, and the mixed weight has a moderate performance, placed between the Gaussian weight and rectangular weight. These results can also be reflected in the coherence curves shown in Figure 6 and Figure 7.

### 5.3. Simulation 2

This simulation shows some scenarios in which the mean of prior distribution is accurate but the variance is smaller or larger. According to the principle of the target filter, a variance of prediction larger than the true variance is common in practice. Meanwhile, an initial given value being inappropriate—or other reasons—may lead to the variance of prediction being overly small; therefore, the mismatch of variance between the prior and true distribution is significant in actual application.

In this simulation, the subarray is based on a ULA which consists of M=200 elements with the element interval of 0.25λ. Here, the required number of array elements is also K=20. This setting is equivalent to adding one uniform spacing element between the array elements in Simulation 1 and keeping the total array aperture the same. The prior distributions are given by $N(500,\sigma_{p}^{2})$ and $N(1500,\sigma_{p}^{2})$, and true distributions are given by $N(500,50^{2})$ and $N(1500,50^{2})$. The other parameters are the same as in Simulation 1.

**Figure 8:**
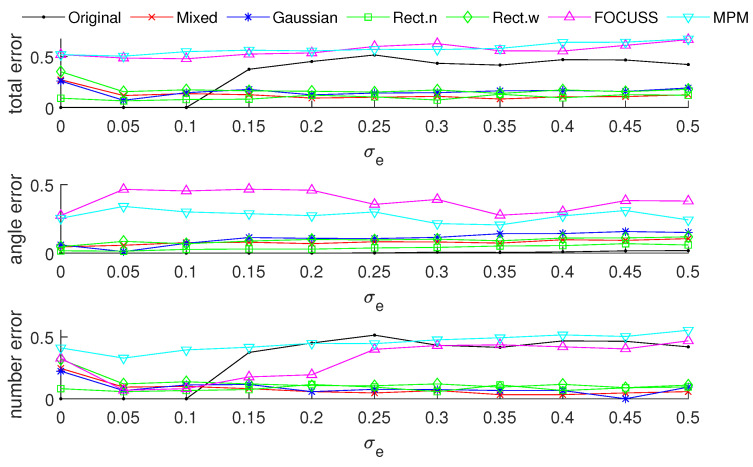
The DOA estimation error in terms of OSPA distance with the prior distribution given by $N(500,50^{2})$ and $N(1500,50^{2})$ when the true distributions are given by $N(500,30^{2})$ and $N(1500,30^{2})$.

**Figure 9:**
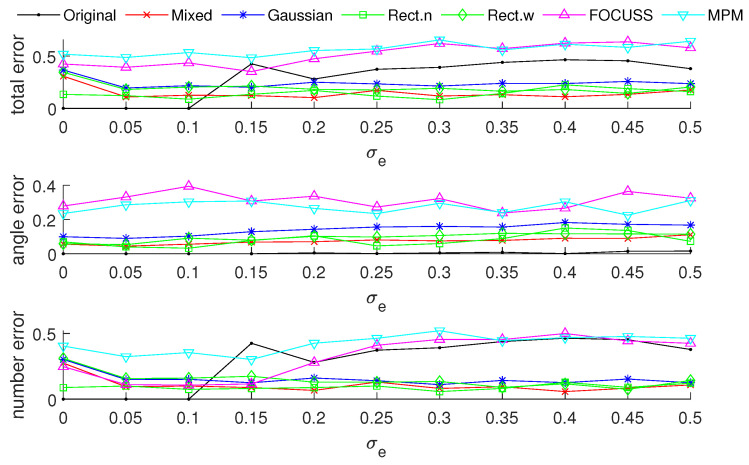
The DOA estimation error in terms of OSPA distance with the prior distribution given by $N(500,50^{2})$ and $N(1500,50^{2})$ when the true distributions are given by $N(500,100^{2})$ and $N(1500,100^{2})$.

**Figure 10:**
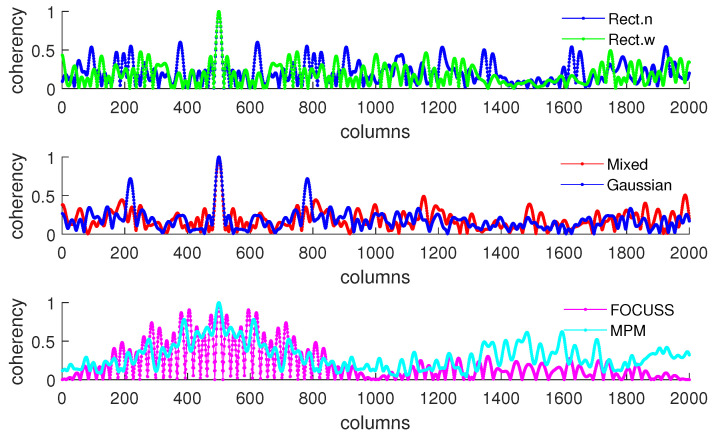
Coherence curves in each subarray generated in Simulation 2 at direction θ=π/6.

**Figure 11:**
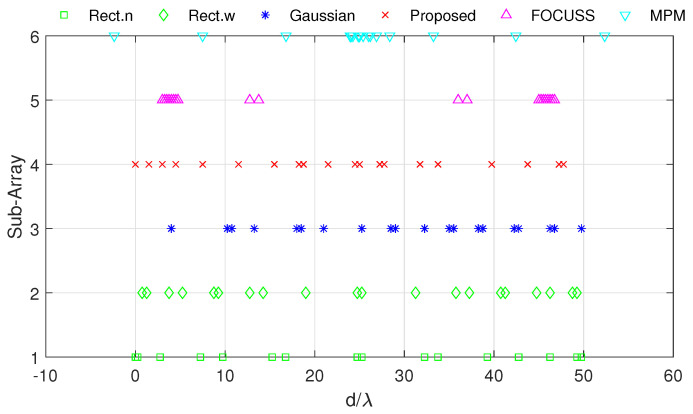
Positions of elements in each subarray generated in Simulation 2.

Figure 8 and Figure 9 show the DOA estimation error in terms of the OSPA distance with the variance of prior distribution given by $\sigma_{p}^{2}=50^{2}$ when the variance of true distribution is given by $\sigma_{t}=30$ and $\sigma_{t}=100$. The coherence curves and element positions of the corresponding subarrays in this simulation are shown in Figure 10 and Figure 11.

Figure 8 is the simulation result under the condition of $\sigma_{p}=50$ and $\sigma_{t}=30$. This scenario corresponds to the case at the beginning of target tracking. In this case, the narrow rectangular weight has the best performance in terms of angle error and total error; the mixed weight performs best in terms of estimating the number of targets, and it has a similar total performance to the narrow rectangular weight.

Figure 9 shows the simulation results under the condition of $\sigma_{p}=50$ and $\sigma_{t}=100$. This scenario corresponds to the case of filter divergence when the measuring system encounters a poor environment. The mixed weight almost has the best performance for the three kinds of errors; the Gaussian wight is worse than the other methods we proposed. In both cases of this simulation, the performance of the FOCUSS method is poor.

### 5.4. Simulation 3

This simulation is performed under the condition of unreliable prior information which completely does not fit the true distribution. The prior distributions of DOAs are given by $N(400,80^{2})$ and $N(800,80^{2})$. The true DOAs, however, are generated from the distributions given by $N(1200,50^{2})$ and $N(1600,50^{2})$.

The original array is a ULA of M=500 elements with an interval of 0.1λ. This setting is equivalent to adding four uniform spacing elements between the array elements in Simulation 1 and keeping the total array aperture the same. The other parameters are the same as Simulation 1.

**Figure 12:**
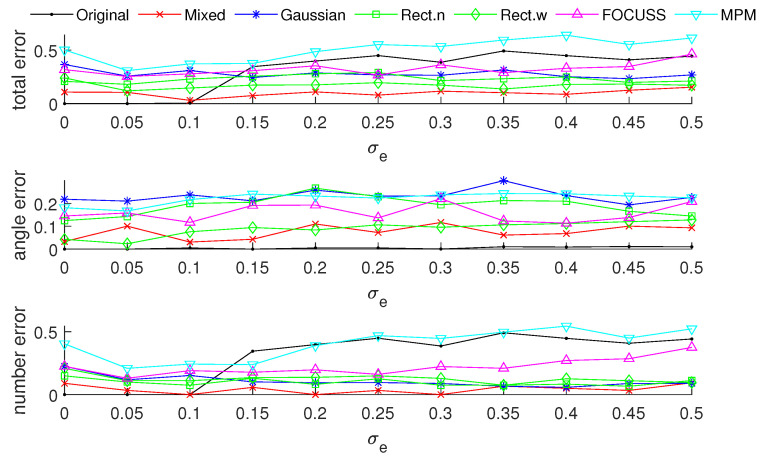
The DOA estimation error in terms of the OSPA distance via different subarrays when the prior distributions are given by $N(400,50^{2})$ and $N(800,50^{2})$ and the true distributions are given by $N(1200,50^{2})$ and $N(1600,50^{2})$.

**Figure 13:**
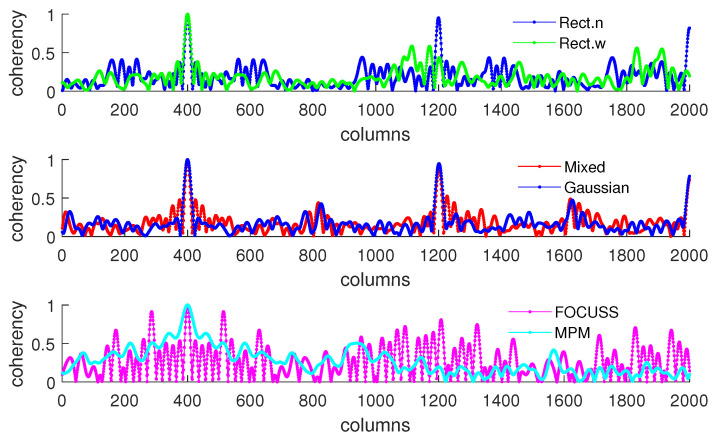
Coherence curves and positions of elements in each subarray generated in Simulation 3 at direction θ=arcsin(0.2).

**Figure 14:**
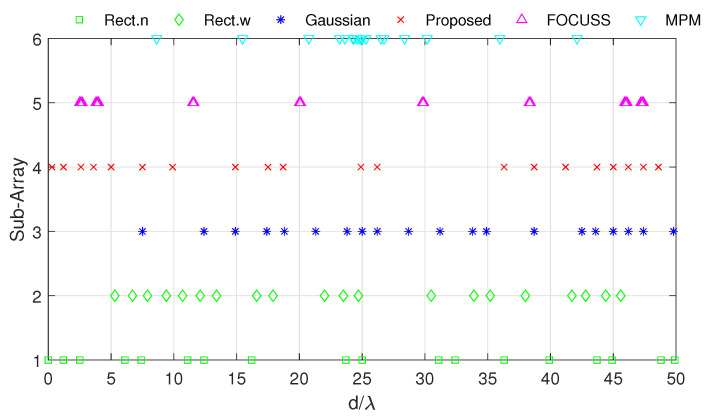
Positions of elements in each subarray generated in Simulation 3.

The DOA estimation error is shown in Figure 12, and the coherence curves at the direction θ=arcsin(0.2) and the element positions of the corresponding subarrays in this simulation are shown in Figure 13 and Figure 14. In this simulation, all the methods have low performance in terms of the mismatch between the prior distribution and the truth. From the perspective of angle error, the Gaussian and narrow rectangular weight methods show poor performance due to their wide main-lobes. For the contrary reason, the mixed weight and wide rectangular weight methods perform well. Consequently, the mixed weight showed the best overall performance in this simulation.

### 5.5. Simulation 4

This simulation is based on a an arbitrary array of 500 elements. Each element location is based on the setting in Simulation 3 but with a random walk which obeys the normal distribution $N(0,0.5^{2})$. The prior distributions of DOAs are given by $N(500,50^{2})$ and $N(1500,50^{2})$. The true DOAs, however, are generated from the distributions given by $N(500,50^{2})$ and $N(1500,50^{2})$. The other settings are the same as Simulation 1.

**Figure 15:**
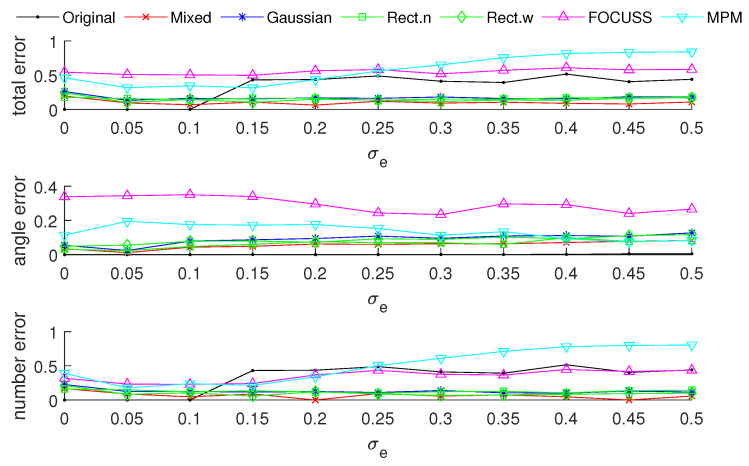
The DOA estimation error in terms of OSPA distance via different subarrays based on an arbitrary array when the prior distribution and the true distribution are both given by $N(500,50^{2})$ and $N(1500,50^{2})$.

**Figure 16:**
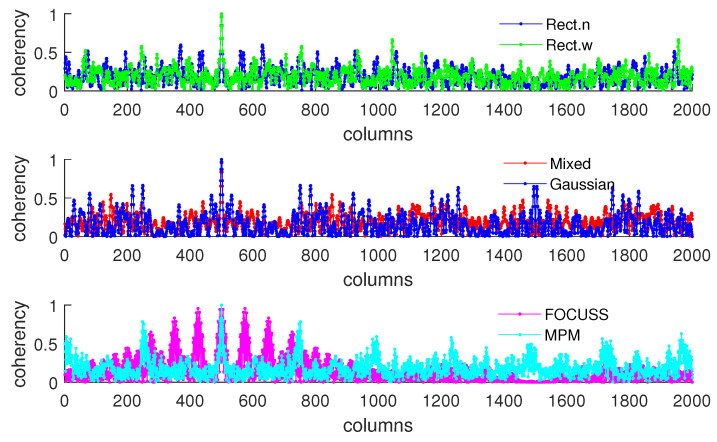
Coherence curves in each subarray generated in Simulation 4 at direction θ=π/6.

**Figure 17:**
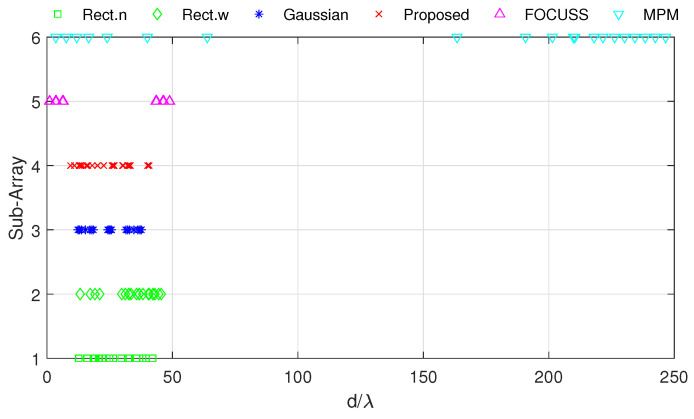
Positions of elements in each subarray generated in Simulation 4.

Figure 15 shows the DOA estimation error in terms of OSPA distance with a variance of prior distribution given by $N(1200,50^{2})$ and $N(1600,50^{2})$ when the true distributions are the same. The coherence curves and element positions of the corresponding subarrays in this simulation are shown in Figure 16 and Figure 17.

Because the original array in this case is equal to the random sampling, which may not meet the requirements of the Shannon–Nyquist theorem, the original array does not have the best performance in all conditions [4]. In contrast, the subarray generated by the proposed algorithm is used for the purpose of optimizing array coherence, while the DOA estimation algorithm used here is based on compressed sensing. Thus, the subarrays generated by the proposed algorithm show better performance.

## 6. Conclusions

In this paper, a subarray selection problem was considered from the perspective of target tracking. In a large actual or virtual array, we selected a portion to form a subarray which was expected to have the closest estimated DOA performance to the original array.

We weighted the sensing matrix by the prior distribution of targets and then obtained the correct singular vectors of the low-rank approximation matrix of the weighted sensing matrix through the SVD technique. The correct singular vectors were considered as the features for subarray selection. Finally, the appropriate elements were selected according to the features. Meanwhile, by analyzing the influence of the weighting type on the singular value and correct singular vectors of the weighted sensing matrix, a mixed type weight was suggested. Through the simulations in different scenarios and the comparison with the relevant algorithms, it can be seen that the proposed method of subarray selection and the suggested mixed weight have good computational performance and can be adapted to different scenarios.

In future work, problems such as array synthesis can be further combined with target filtering, and the demand of the target filter at different times can be taken as the object of array synthesis in order to obtain optimal overall performance.

## Figures and Tables

**Table 1:** The runtime of each method in Simulation 1.

Method	Effective Width	Number of Alternative Elements (*M*)	Runtime/s
Narrow Rectangular	202	100	0.0054
Wide Rectangular	404	100	0.0102
Gaussian	742	100	0.0266
Mixed	770	100	0.0316
FOCUSS	202	100	0.0352
MPM	202	100	0.0027

**Table 2:** The runtime of each method in Simulation 4.

Method	Effective Width	Number of Alternative Elements (*M*)	Runtime/s
Narrow Rectangular	202	500	0.0332
Wide Rectangular	404	500	0.0661
Gaussian	742	500	0.1329
Mixed	770	500	0.1415
FOCUSS	202	500	0.1779
MPM	202	500	0.0568
